# Generation and analysis of ESTs from the eastern oyster, *Crassostrea virginica *Gmelin and identification of microsatellite and SNP markers

**DOI:** 10.1186/1471-2164-8-157

**Published:** 2007-06-08

**Authors:** Jonas Quilang, Shaolin Wang, Ping Li, Jason Abernathy, Eric Peatman, Yongping Wang, Lingling Wang, Yaohua Shi, Richard Wallace, Ximing Guo, Zhanjiang Liu

**Affiliations:** 1 The Fish Molecular Genetics and Biotechnology Laboratory, Department of Fisheries and Allied Aquacultures and Program of Cell and Molecular Biosciences, Aquatic Genomics Unit, 203 Swingle Hall, Auburn University, Auburn, AL 36849, USA; 2 Institute of Biology, College of Science, University of the Philippines, 1101 Diliman, Quezon City, Philippines; 3 Haskin Shellfish Research Laboratory, Institute of Marine and Coastal Sciences, Rutgers University, 6959 Miller Ave., Port Norris, NJ 08349, USA

## Abstract

**Background:**

The eastern oyster, *Crassostrea virginica *(Gmelin 1791), is an economically important species cultured in many areas in North America. It is also ecologically important because of the impact of its filter feeding behaviour on water quality. Populations of *C. virginica *have been threatened by overfishing, habitat degradation, and diseases. Through genome research, strategies are being developed to reverse its population decline. However, large-scale expressed sequence tag (EST) resources have been lacking for this species. Efficient generation of EST resources from this species has been hindered by a high redundancy of transcripts. The objectives of this study were to construct a normalized cDNA library for efficient EST analysis, to generate thousands of ESTs, and to analyze the ESTs for microsatellites and potential single nucleotide polymorphisms (SNPs).

**Results:**

A normalized and subtracted *C. virginica *cDNA library was constructed from pooled RNA isolated from hemocytes, mantle, gill, gonad and digestive tract, muscle, and a whole juvenile oyster. A total of 6,528 clones were sequenced from this library generating 5,542 high-quality EST sequences. Cluster analysis indicated the presence of 635 contigs and 4,053 singletons, generating a total of 4,688 unique sequences. About 46% (2,174) of the unique ESTs had significant hits (E-value ≤ 1e-05) to the non-redundant protein database; 1,104 of which were annotated using Gene Ontology (GO) terms. A total of 35 microsatellites were identified from the ESTs, with 18 having sufficient flanking sequences for primer design. A total of 6,533 putative SNPs were also identified using all existing and the newly generated EST resources of the eastern oysters.

**Conclusion:**

A high quality normalized cDNA library was constructed. A total of 5,542 ESTs were generated representing 4,688 unique sequences. Putative microsatellite and SNP markers were identified. These genome resources provide the material basis for future microarray development, marker validation, and genetic linkage and QTL analysis.

## Background

The eastern oyster, *Crassostrea virginica *(Gmelin, 1791) occurs naturally in the Western Atlantic from the Gulf of St. Lawrence in Canada to the Gulf of Mexico, Caribbean, and coasts of Brazil and Argentina [1]. This species has been introduced to the Pacific coast of North America, Europe, and Hawaii but it has maintained reproducing populations in only two localities outside its natural range, namely, in a river in British Columbia and in a small basin in Hawaii [2]. It thrives in estuaries and coastal areas and is present in some areas as extensive reefs. These reefs are vital components of estuarine ecosystems because they not only serve as habitat for many fishes but also as a refuge for them and for some reef-associated invertebrates when environmental conditions become stressful [3]. The eastern oyster is an economically important species in North America as natural populations are being harvested extensively. It is also being cultivated in many areas. The eastern oyster populations have been threatened by overfishing and habitat degradation as a result of expanding utilization of coastlines and other anthropogenic disturbances [3,4]. Outbreak of major diseases such as Dermo (caused by a protozoan pathogen, *Perkinsus marinus*) and MSX (Multinucleate Sphere X, caused by another protozoan *Haplosporidium nelsoni*) have devastated both farmed and wild populations of the oyster species.

Oysters are a fundamental component of the aquatic ecosystem in addition to their importance to fisheries and aquaculture industries. As filter-feeding bivalves, oysters play a critical role in maintaining water quality [5]. The eastern oyster has also been used as a marine bivalve model to study the effects of environmental stressors [6] and as bioindicators of estuarine pollution [7,8]. A number of genome resources have been developed from the eastern oyster including construction of a framework genetic linkage map [9], construction of large-insert BAC libraries [10], initial analysis of expressed sequence tags (EST) [11-13], and construction of the first generation microarray [14].

EST analysis is not only the most efficient approach for gene discovery, but also an effective approach for the identification of polymorphic DNA markers such as microsatellites and single nucleotide polymorphisms [15,16] that are highly useful for genetic mapping and comparative genome analysis. However, EST analysis in the eastern oysters has been hindered by highly abundantly expressed genes in oysters including a very high proportion of mitochondrial genes [12,17]. Compared to the very large efforts for the development of EST resources for the Pacific oysters ([18,19], and the ongoing efforts at the Joint Genome Institute), the EST resource in the eastern oyster is small. To date, only 9,018 ESTs have been deposited to GenBank from *C. virginica*. Due to high redundancy rate of the oyster ESTs, the unique genes represented by these ESTs are relatively small. The objectives of this study were to construct a normalized cDNA library for efficient generation of unique ESTs from the eastern oysters, and to generate additional ESTs from the normalized cDNA library. Here we report the construction of a high-quality normalized cDNA library and the generation and analysis of 5,542 ESTs.

The number of microsatellite markers available for mapping has been low. To date, there are about 104 microsatellite markers developed from the eastern oyster. Seven (7) polymorphic microsatellite markers were developed from a size-selected (400 – 900 bp) partial genome library [20]. Eighteen (18) microsatellite markers were developed by screening 743 clone sequences for microsatellite repeats from a small-insert *C. virginica *genomic library [21]. Sixty one (61) EST-linked microsatellite markers were developed by screening eastern oyster sequences from public databases [22,23]. Three (3) microsatellites were isolated from *C. virginica *genomic DNA [24]. Eleven (11) microsatellites were originally developed from *C. gigas *which also yielded cross-species amplifications in *C. virginica *[25,26]. The remaining four microsatellite markers were developed by the Haskin Shellfish Research laboratory at Rutgers University, USA [27]. In this work, we have identified 35 microsatellites within the sequenced ESTs, of which 18 have sufficient flanking sequences for primer design. Using the sequenced ESTs, along with those ESTs already existing in the GenBank, we identified 6,533 single nucleotide polymorphic sites that are potentially useful for genetic linkage mapping and QTL analysis in this species.

## Results

### Generation of ESTs and contig assembly

A total of 6,528 randomly picked EST clones were sequenced, producing 6,349 readable sequences (97.3% sequencing success rate). After removal of clones with no inserts or very short inserts (100 bp cutoff), a total of 5,542 high-quality ESTs (Table 1) were produced with an average length of 473 bp. The cumulative length of all high-quality EST sequences was 2,622,701 bases. All the ESTs have been deposited to GenBank with continuous accession numbers of EH643873 – EH649414.

**Table 1:** Summary Statistics of ESTs generated from the eastern oyster, *C. virginica*

Feature	Value
Total number of clones sequenced	6,528
Number of high-quality ESTs	5,542
Average length of high quality ESTs (bp)	473
Number of contigs	635
Number of ESTs in contigs	1,489
Number of singletons	4,053
Number of unique sequences	4,688

The ESTs were subjected to cluster analysis using three software, PHRAP, CAP3, and Vector *NTI Advance*™ 10, with the final contigs assembled with Vector *NTI Advance*™ 10. A total of 635 contigs were assembled consisting of 1,489 ESTs while 4,053 sequences were singletons (Table 1). Thus, this EST project allowed identification of 4,688 unique sequences. Of the 635 contigs, 500 contained 2 ESTs (78.7%); 108 contained 3 ESTs (17%); 19 contained 4 ESTs; four contained 5 ESTs, three contained 6 ESTs; and one contained 51 ESTs (Table 2). Clearly, most of the clusters were small, a reflection of high efficiency in normalization and subtraction. The single large cluster was from mitochondrial sequences, and that could have been missed in the subtraction.

**Table 2:** Statistics of BLASTX searches

Number of unique sequences	4,688
Number of unique sequences with BLASTX hits	2,174
Percentage of unique sequences with BLASTX hits	46.4
Number of contigs containing	
2 ESTs	500
3 ESTs	108
4 ESTs	19
5 ESTs (homologues of actin, tubulin, and two unknown genes)	4
6 ESTs (all unknown)	3
51 ESTs (homologous to oyster mitochondrial genome)	1

The ESTs generated from this study were searched against ESTs of *C. virginica *in dbEST using BLASTN. Of the 4,688 unique sequences generated in this study, 901 (19.2%) had significant hits (*e *≤ 10^-5^) with existing ESTs from *C. virginica*, while 3,787 (80.8%) were found to be novel *C. virginica *ESTs. Therefore, this EST collection represented a significant addition to the existing oyster EST resources.

### Putative identities of the ESTs

**Figure 1:**
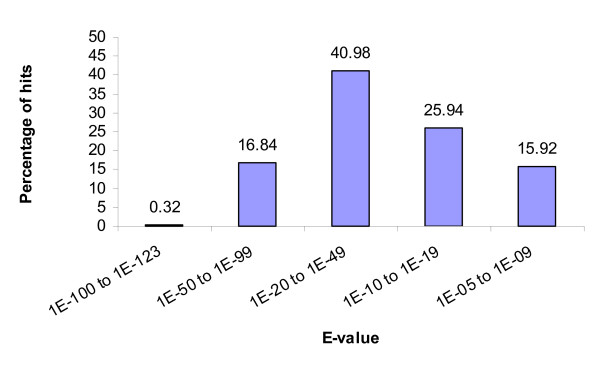
Distribution of E-values from the top hit in the non-redundant protein database.

**Figure 2:**
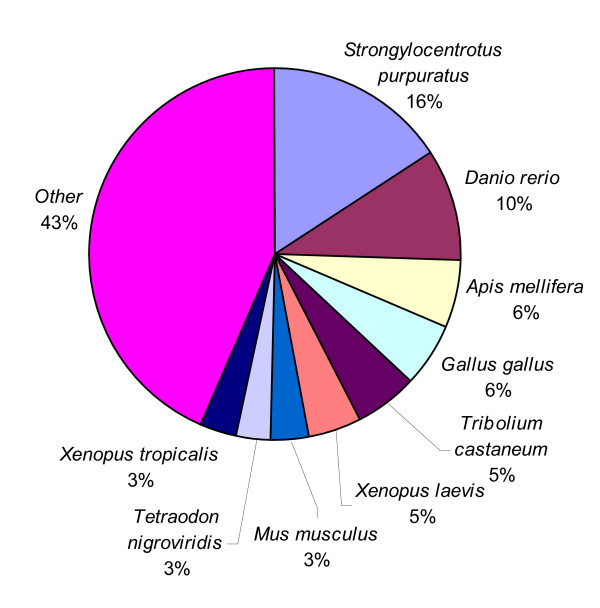
**Organisms with the most protein hits**. The proportion of putative transcripts with BLASTX matches (E-value ≤ 10^-5^) in the non-redundant protein database classified according to the organism of the "top hit" protein sequence.

In order to make an assessment for the putative identities of the sequenced ESTs, all ESTs were subjected to BLASTX similarity searches. Of the 4,688 unique sequences, 2,174 (46.4%) had significant matches (E ≤ 10^-5^) to the non-redundant (nr) protein database using BLASTX (Additional file 1). Of the unique sequences with matched homologies in the nr protein database, seven (0.32 %) had an E-value of 1e-100 or less (Figure 1) and are therefore considered highly significant homology [28]. About 58% had significant homology (E-values between 1e-20 and 1e-99) and thus, are considered moderately similar [28,29]. About 42% of the ESTs were assigned weak homology (E-values between 1e-05 and 1e-19) and are therefore weakly similar. Among the various organisms that have protein sequences in GenBank, the *C. virginica *ESTs generated from this study had the highest number of BLASTX hits to the purple sea urchin *Strongylocentrotus purpuratus *(16%), followed by zebrafish *Danio rerio *(10%), honey bee *Apis melifera *(6%), and chicken *Gallus gallus *(6%) (Figure 2). Among the organisms listed in "other" (43% of total), 13 ESTs (0.6 %) had significant hits to *C. virginica *proteins in the database and 35 ESTs (1.6%) had hits with the Pacific oyster, *Crassostrea gigas*.

Of the putatively identified genes, 29 different putative proteins were related to stress response, 10 were related to immune response, and 13 were related to defense responses (Table 3). These genes are of interest for further studies in relation to disease resistance, and stress responses of oysters, a significant issue in oyster industries.

**Table 3:** *C. virginica *ESTs similar to genes potentially involved in stress response, defense mechanisms and immune response

**GenBank Accession no.**	**Putative match**	**Homolog species**	**E-value**	**Hit ID**
***Stress response***				
Contig\345 (EH646797 &EH645944)	84 kDa heat shock protein	*Haliotis tuberculata*	7E-78	CAK95235.1
EH646306	CCAAT/enhancer binding protein (C/EBP), gamma	*Danio rerio*	2E-17	NP_571961.1
EH645147	C-type lectin 2	*Anguilla japonica*	5E-19	BAC54021.1
EH647492	ecto-ATP diphosphohydrolase I	*Homo sapiens*	1E-07	CAB41886.1
Contig\100 (EH644789 &EH646960)	ENSANGP00000017916 (belongs to the ubiquitin-conjugating enzyme family)	*Aedes aegypti*	2E-65	XP_310416.4
EH645340	Flap endonuclease 1-B	*Xenopus laevis*	2E-72	P70054
EH646336	growth arrest and DNA damage 45 gamma like	*Danio rerio*	7E-10	NP_998391.1
Contig\310 (EH646499 &EH647053)	*Homo sapiens *ubiquitin-conjugating enzyme E2N	synthetic construct	2E-69	AAP36228.1
EH644486	MGC84195 protein	*Xenopus laevis*	8E-29	AAH82452.1
EH646608	Mn superoxide dismutase	*Biomphalaria glabrata*	3E-49	AAS83980.1
EH648076	novel protein similar to vertebrate methionine sulfoxide reductase A	*Danio rerio*	9E-35	CAH68999.1
Contig\517 (EH648399 &EH648432)	Peroxiredoxin V protein	*Branchiostoma belcheri*	4E-58	AAM18076.1
EH645663	PREDICTED: E1A binding protein p300 isoform 2	*Pan troglodytes*	6E-31	XP_001168473.1
EH645265 and EH646835	PREDICTED: hypothetical protein	*Strongylocentrotus purpuratus*	6E-37	XP_001189673.1
EH646242	PREDICTED: hypothetical protein isoform 2	*Gallus gallus*	1E-07	XP_421849.1
EH645065	PREDICTED: similar to collectin sub-family member 12 isoform II	*Macaca mulatta*	6E-10	XP_001088438.1
EH645286	PREDICTED: similar to cryptochrome 1 (photolyase-like)	*Tribolium castaneum*	1E-39	XP_972654.1
Contig\578 (EH648875 &EH648870)	PREDICTED: similar to Der1-like domain family, member 1	*Strongylocentrotus purpuratus*	2E-70	XP_797383.1
EH646861	PREDICTED: similar to dsRNA adenosine deaminase	*Strongylocentrotus purpuratus*	3E-16	XP_001183590.1
EH646254	PREDICTED: similar to ER degradation-enhancing alpha-mannosidase-like	*Canis familiaris*	6E-58	XP_533753.2
EH646603	PREDICTED: similar to GTP-binding-protein CG5519-PA	*Apis mellifera*	6E-61	XP_392284.2
EH646719	PREDICTED: similar to mitogen-activated protein kinase kinase 7	*Apis mellifera*	1E-56	XP_396834.1
EH648858	PREDICTED: similar to oxidative stress protein	*Strongylocentrotus purpuratus*	1E-12	XP_001178682.1
EH647765	PREDICTED: similar to scavenger receptor cysteine-rich protein type 12 precursor	*Strongylocentrotus purpuratus*	8E-19	XP_791976.2
EH648904	PREDICTED: similar to stress-induced-phosphoprotein 1	*Tribolium castaneum*	5E-44	XP_967038.1
EH646424	ring finger protein 7	*Gallus gallus*	8E-47	NP_001026478.1
EH645238	Unknown	*Branchiostoma floridae*	3E-15	AAM18895.1
EH647041	unnamed protein product	*Tetraodon nigroviridis*	2E-09	CAG05929.1
EH648535	Zgc:153458	*Danio rerio*	1E-26	AAI24360.1
***Immune response***				
EH647765	PREDICTED: similar to scavenger receptor cysteine-rich protein type 12 precursor, partial	*Strongylocentrotus purpuratus*	8E-19	XP_791976.2
EH645065	PREDICTED: similar to collectin sub-family member 12 isoform II	*Macaca mulatta*	6E-10	XP_001088438.1
EH644243	peptidoglycan recognition protein	*Biomphalaria glabrata*	4E-10	ABK76644.1
EH646861	PREDICTED: similar to dsRNA adenosine deaminase	*Strongylocentrotus purpuratus*	3E-16	XP_001183590.1
EH645147	C-type lectin 2	*Anguilla japonica*	5E-19	BAC54021.1
EH646306	CCAAT/enhancer binding protein (C/EBP), gamma	*Danio rerio*	2E-17	NP_571961.1
Contig\188 (EH645432 &EH647545)	PREDICTED: leukocyte-derived arginine aminopeptidase isoform 3	*Pan troglodytes*	4E-28	XP_001138283.1
EH648526	PREDICTED: inhibitor of kappa light polypeptide gene enhancer in B-cells, kinase complex-associated protein isoform 1	*Pan troglodytes*	4E-34	XP_001142759.1
Contig\517 (EH648399 &EH648432)	peroxiredoxin V protein	*Branchiostoma belcheri*	4E-58	AAM18076.1
EH644486	MGC84195 protein	*Xenopus laevis*	8E-29	AAH82452.1
***Defense response***				
EH646306	CCAAT/enhancer binding protein (C/EBP), gamma	*Danio rerio*	2E-17	NP_571961.1
EH645147	C-type lectin 2	*Anguilla japonica*	5E-19	BAC54021.1
EH645566	Defensin	*Crassostrea gigas*	2E-16	CAJ19280.1
EH645733	Lysozyme	*Crassostrea virginica*	3E-72	BAE47520.1
EH644486	MGC84195 protein	*Xenopus laevis*	8E-29	AAH82452.1
EH644243	Peptidoglycan recognition protein	*Biomphalaria glabrata*	4E-10	ABK76644.1
EH645480 &EH649320	Peptidoglycan recognition protein S1 precursor	*Chlamys farreri*	1E-25	AAY53765.1
Contig\517 (EH648399 &EH648432)	Peroxiredoxin V protein	*Branchiostoma belcheri*	4E-58	AAM18076.1
EH648526	PREDICTED: inhibitor of kappa light polypeptide gene enhancer in B-cells	*Pan troglodytes*	4E-34	XP_001142759.1
Contig\188 (EH645432 &EH647545)	PREDICTED: leukocyte-derived arginine aminopeptidase isoform 3	*Pan troglodytes*	4E-28	XP_001138283.1
EH645065	PREDICTED: similar to collectin sub-family member 12 isoform II	*Macaca mulatta*	6E-10	XP_001088438.1
EH646861	PREDICTED: similar to dsRNA adenosine deaminase	*Strongylocentrotus purpuratus*	3E-16	XP_001183590.1
EH647765	PREDICTED: similar to scavenger receptor cysteine-rich protein type 12 precursor	*Strongylocentrotus purpuratus*	8E-19	XP_791976.2

Of the 2,514 unique sequences without significant matches to the nr protein database, 6 (includes 4 contigs consisting of 57 ESTs and 2 singletons) had matches using the BLASTN algorithm (E-value ≤ e-5). All of these correspond to mtDNA sequences.

### Gene ontology annotation

**Figure 3:**
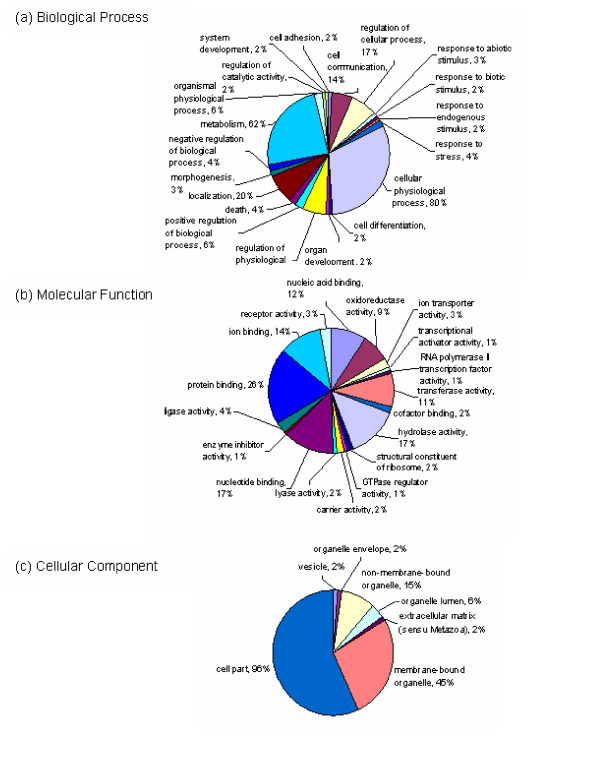
**Gene Ontology (GO) assignment (3^rd ^level GO terms) of 1,104 *C. virginica *annotated ESTs**. The total numbers of ESTs annotated for each main category are 761 for Biological Process, 952 for Molecular Function, and 614 for Cellular Component. Since a gene product could be assigned to more than one GO term, the percentages in each main category do not add up to 100%.

Gene Ontology (GO) categories were assigned to 1,104 unique ESTs with BLASTX hits using Blast2GO. Figure 3 shows the percentage distributions of gene ontology terms (3^rd ^level GO terms) according to the GO consortium. Cellular physiological process (80 %) was the most dominant 3^rd ^level term out of the 761 unique sequences which were annotated to the Biological Process GO category. This was followed by metabolism at 62%. Only 4% were assigned to the Biological Process subcategory response to stress. Protein binding (26%) was the most dominant out of 952 ESTs with significant protein hits which were assigned to Molecular Function category at 3^rd ^level. This was followed by hydrolase activity and nucleotide binding at 17% each. GO terms at higher and lower levels for each of the three main GO categories as well as the unique ESTs which fall on each term are given in additional file 2.

### Microsatellites-containing ESTs

Thirty-five (35) microsatellites were found in 32 of the 4,688 unique EST sequences using Msatfinder. The major types of the identified microsatellites were dinucleotide (17) and trinucleotide (16) (Table 4). Of the 32 unique ESTs with microsatellites, 17 have sufficient flanking sequences for the design of 18 primers, and are therefore potentially useful for genetic linkage mapping. Two (2) of the 32 microsatellite-containing ESTs have significant matches to the nr database. These are contig 97 (GenBank accession nos. EH644779 and EH646080) which is homologous to an unnamed protein product in *Tetraodon nigroviridis *and contig 366 (GenBank accession nos. EH647022 and EH644181) which is homologous to a predicted protein similar to axonemal leucine-rich repeat protein in *Strongylocentrotus purpuratus*. Of the microsatellites containing ESTs, two contigs were assembled. *In silico *comparison of the sequences included in the two contigs indicated that the one microsatellite was polymorphic while the other microsatellite was not polymorphic between the two sequenced ESTs.

**Table 4:** Microsatellite-containing ESTs with available designed primers using Msatfinder

**GenBank Accession no.**	**Length (bp)**	**Repeat motif**	**No. of repeats**
EH644527	363	AGG	7
EH644818	580	TC	17
EH645930	655	GTG	7
EH646768	629	CAA	10
EH647168	561	GAG	6
EH647292	528	AAC	7
EH647319	477	ATTG	6
EH647493	376	GA	14
EH647621	657	AAG	6
EH647703	423	AG	18
EH647704	643	TC	13
EH647722	634	AGA	7
EH647722	634	GAA	14
EH647780	316	AAC	8
EH647816	434	TG	8
EH648299	616	AG	22
EH648644	534	CAA	6
EH649350	509	GAA	8

### Single nucleotide polymorphism

Single nucleotide polymorphism (SNP) has recently become the marker type of choice for linkage and QTL analysis. In most cases, SNP discovery has relied on genomic sequencing, BAC end sequencing, or targeted SNP discovery [30-33]. In the eastern oyster, there have been no reports on large-scale SNP identification. In this study, we took advantage of the existing ESTs with high redundancy along with the ESTs reported here. A total of 1,486 contigs were assembled including 7,702 oyster EST sequences. A total of 6,533 putative SNPs were identified including 2,528 transitions, 1,933 transversions, and 2,072 indels (Table 5). These SNPs represented a rate of 0.59 SNP per 100 base pairs. However, as the quality scores were not available for all ESTs used in the analysis, the putative SNPs could have been caused by sequencing errors as well as the true SNPs. In order to provide some assessment of the SNPs, the putative SNPs were categorized based on the contig sizes. Clearly, the larger the number of sequences involved in a contig, the more likely the SNPs can be checked as to whether the putative SNPs represent sequence errors or real SNPs. As shown in Table 6, 2,108 (32.3%) putative SNPs were identified from contigs with only two sequences; 1,856 (28.4%) putative SNPs were identified from contigs with three sequences; 1,580 (24.2%) putative SNPs were identified from contigs with four sequences; and 989 (15.1%) putative SNPs were identified from contigs with five or more sequences (Table 6).

**Table 5:** Summary of putative SNP identification from oyster EST resources

Total sequences analysed	7702
Number of contigs	1486
Total SNPs detected	6533
SNP frequency	0.59/100 bp
Total number of transitions	2528
Total number of transversions	1933
Total number of Indels	2072

**Table 6:** Putative SNP distribution in contigs with various numbers of ESTs

	Number of contigs	Putative SNP sites
with > 50 sequences	7	389
with 11–50 sequences	70	171
with 6–10 sequences	132	253
with 5 sequences	74	176
with 4 sequences	129	1580
with 3 sequences	262	1856
with 2 sequences	812	2108
Total	1486	6533

## Discussion

**Figure 4:**
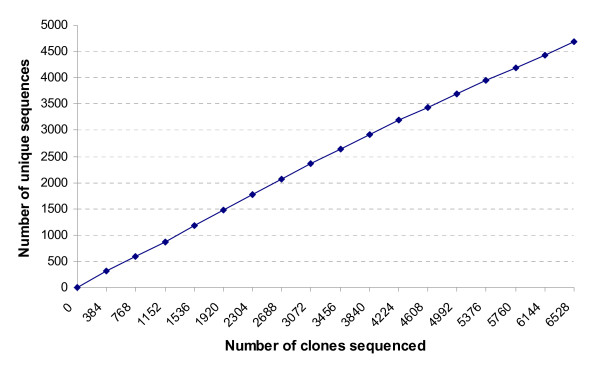
**Low redundancy of *C. virginica *ESTs**. Number of unique ESTs were plotted as a function of the total number of clones sequenced. Note that the relationship was nearly linear, suggesting a high rate of gene discovery.

This work produced a total of 5,542 ESTs representing 4,688 unique sequences. This EST collection represents the largest number of unique sequences from the eastern oysters. Previous efforts in EST sequencing from this species revealed a high level of sequencing redundancy, largely due to very high proportion of mitochondrial genes [12,17]. In this work, the cDNA library was normalized, along with subtraction of the most abundantly expressed genes as determined from previous EST sequencing efforts. Cluster analysis indicated that only one contig was large representing mitochondrial sequences. For the most part, however, the normalized/subtracted cDNA library allowed efficient generation of unique EST sequences. This suggested that not only normalization, but also subtraction is required for the construction of cDNA libraries suitable for large-scale EST analysis in oysters. Our EST analysis here had a gene discovery rate of 84.6%, or a redundancy rate of 15.4%. Clearly, this is a reflection of the quality of the normalized library. As shown in Figure 4, after sequencing of 6,000 some clones, the rate of gene discovery was still in the linear phase in relation to the number of sequenced clones. This warrants the use of this normalized cDNA library for additional EST sequencing when funding situation permits.

Similarity searches of the newly generated ESTs against the existing ESTs in the dbEST revealed that of the 4,688 unique sequences generated in this study, only 901 (19.2%) had significant hits with existing ESTs from *C. virginica*, while 3,787 (80.8%) were found to be novel *C. virginica *ESTs. Therefore, this EST collection represented a significant addition to the existing oyster EST resources. These novel ESTs should provide the material basis for the new editions of the oyster microarrays.

The high percentage of *C. virginica *ESTs (54% in this study and in [12]) with no hits in the protein database implies that there is an enormous potential for discovery of new genes in this organism and possibly new gene networks and metabolic pathways [34]. However, we realize that the ESTs were relatively short, and many of the ESTs could still be within the 5' untranslated region (UTR) such that their identities could not be revealed by sequence similarity comparisons. Efforts should be made in the generation of complete cDNA sequences in this species in order to provide a greater level of assessment of its gene contents and similarities to various other species in the evolutionary spectrum.

A number of stress-, immune-, and defense-related transcripts were putatively identified from this study (e.g., Table 3). These genes are of particular interest to oyster researchers because of relentless environmental pressure on natural and farmed oyster populations brought about by the increasing use of coastal zones and also because of the devastating effects of diseases [11-13]. In this study, GO annotation of sequences with protein matches in nr database identified 29 distinct transcripts which are potentially involved in stress responses, 10 distinct transcripts that are potentially involved in immune responses, and 13 distinct transcripts that are potentially involved in defense responses (Table 3). While many of these genes were reported in previous studies on *C. virginica *ESTs [11-13], additional studies of these genes are needed to determine their expression in relation to stresses or diseases in order to develop molecular indicators for environmental stresses.

Among the myriad of applications of ESTs is the development of microsatellite and SNP markers. This work allowed the identification of 32 microsatellite-containing ESTs, of which 18 had sufficient flanking sequences for primer design. These microsatellites should be useful for genetic linkage mapping in this species. However, polymorphism needs to be tested in specific resource families. A large number (6,533) of putative SNPs were identified from the overlapping sequences of ESTs. These putative SNPs are potentially useful for genetic linkage mapping and for the analysis of quantitative traits of the eastern oysters. However, validation and polymorphic analysis must be performed before these putative SNPs can be used. That is because a large proportion of these putative SNPs were identified from contigs with just two or three sequences. In the absence of the quality scores, it was not possible for us to differentiate the true SNPs from sequence errors. In spite of this setback, the putative SNPs identified from this study represent the first large set of SNPs from the eastern oysters. With additional validation, as well as testing in specific resource families for their polymorphism will make them useful markers for genetic analysis in oysters. In a recent study, over 80% of the SNPs derived from GenBank sequences have been validated as true SNPs in the eastern oyster [35].

## Conclusion

A high-quality normalized/subtracted cDNA library was constructed as determined by the high gene discovery rate (84.6%) with over 6,000 clones of ESTs being sequenced. We have generated 5,542 ESTs representing 4,688 unique sequences. Of the 4,688 unique sequences, the majority (3,787) were novel ESTs for this species. Therefore, this EST collection represents a significant addition to the existing EST resources of the eastern oysters. A good fraction (46%) of the unique ESTs had significant similarities to known sequences in GenBank. These ESTs should serve as valuable resources for the development of microarrays useful for functional genomic analysis. In addition to the use as EST resources, we have also identified putative microsatellite and SNP markers. Once validated and tested for polymorphism, these microsatellite and SNP markers should be useful for genetic linkage and QTL analysis in oysters.

## Methods

### Tissue source and RNA isolation

Oysters from Rutgers University (NEHY-53) were used in this study. Samples of tissues (50 mg from 4 oysters for each tissue type) were excised from gill, mantle, digestive tract and gonad, and adductor muscle. In addition, about 100 mg of tissue was obtained from one whole juvenile oyster and hemocytes were extracted from 12 oysters. Total RNA was prepared from each sample by using RNeasy Mini kits (Qiagen, Valencia, CA). Total RNA quality and quantity were checked by agarose gel electrophoresis containing formaldehyde and by using a spectrophotometer. Equal amounts of total RNA from each sample listed above were mixed for cDNA library construction.

### Construction of normalized and subtracted cDNA library

The Creator™ SMART™ cDNA Library Construction Kit (Clontech, Mountain View, CA) and components from the TRIMMER-DIRECT Kit from Evrogen (Moscow, Russia) were used for the construction of the normalized cDNA library. One microgram (1 μg) of mixed total RNA was combined with 1 μl of SMART IV oligonucleotide (Clontech) and 1 μl CDS-3 M adapter (Evrogen) for first-strand cDNA synthesis. The reaction was incubated at 72°C for 2 min followed by immediate cooling on ice for 2 min. Then, 2 μl of 5X first strand buffer, 1 μl of DTT (20 mM), 1 μl of dNTP mix (10 mM), and 1 μl of PowerScript reverse transcriptase were added to the tube and incubated at 42°C for 1 hr in a thermal cycler (PTC-100, Bio-Rad, Hercules, CA) and placed on ice. The SMART cDNA cloning system allows the enrichment of full-length cDNA through the use of a 5'-linker with 3'-GGG tails. Reverse transcriptase has terminal transferase activity that preferentially adds three additional Cs at the end of first strand cDNA. As a result, the first strand cDNA is able to base pair with the 5'-linker with 3'-GGG tails. Once base paired, the reverse transcriptase switches templates and extends into the linker sequences allowing PCR amplification of full-length cDNA using a single primer (the 5'-linker has the same sequences as the linker containing poly-T used for the synthesis of the first strand cDNA). Truncated cDNAs are not able to base pair with the 5'-linker and, therefore, get lost in the PCR amplification of the full-length cDNA. The first strand cDNA was initially amplified by long-distance PCR (LD-PCR) using hot-start amplification. For the reaction, the following were combined in a reaction tube: 1.5 μl of the first strand cDNA, 60 μl of sterile deionized water, 7.5 μl of 10X Advantage 2 PCR buffer, 1.5 μl of 50X dNTP mix, 3 μl of 5' PCR primer and 1.5 μl of 50X Advantage 2 polymerase mix. The tube was mixed and briefly centrifuged and added to a pre-heated (95°C) thermal cycler. Cycle settings were 95°C for 1 min followed by 19 cycles of 95°C for 7 sec, 66°C for 20 sec, and 72°C for 5.5 min. The product was analyzed on a 1.1% agarose gel to determine the sizes and amount of the cDNA products before proceeding to the next step. The LD-PCR reaction was purified and eluted in 30μl of sterile nanopure water using the QIAquick PCR Purification Kit (Qiagen). For the normalization procedure, the TRIMMER-DIRECT Kit from Evrogen (Moscow, Russia) was used. This system is specially developed to normalize cDNA enriched with full length sequences. The cDNA from the LD-PCR was quantified (~200 ng/μl) and 1 μl was mixed with 1 μl of 4X hybridization buffer, 1 μl of sterile water, and 1 μl (~500 ng/μl) of PCR fragments of 135 abundant ESTs (see additional file 3 for the list) from a previous oyster cDNA library developed in our lab [12]. The mix was overlaid with mineral oil and incubated for 3 min at 98°C then at 70°C for 4 hr. Then, 5 μl of 2X DSN buffer (preheated to 70°C) and 0.25 Kunitz units of DSN enzyme were added and incubated at 70°C for 20 min. The DSN enzyme specifically degrades double-stranded molecules. The reaction was inactivated by adding 10 μl of DSN stop solution, and sterile water added to a final volume of 40 μl. Following normalization, two rounds of PCR were performed using 1 μl of the normalization reaction as template. A shorter primer M1 (first 23 bases of the SMART IV oligonucleotide) was used in the first round of PCR with 15 amplification cycles using the same thermal cycling parameters as above; and an even shorter primer M2 (first 20 bases of the SMART IV oligonucleotide) was used in the second round of PCR for 15 amplification cycles of 95°C for 7 sec, 64°C for 20 sec, and 72°C for 5.5 min. Products were checked on a 1.1% agarose gel. The PCR products were quantified and 3 μg were used for treatment with proteinase K. All the subsequent procedures including proteinase K treatment, restriction digestion with *Sfi *I, size fractionation, and ligation followed the manufacturer's instructions (Clontech). The cDNA was ligated to the pDNR-LIB vector. Electroporation (MicroPulser, Bio-Rad, Hercules, CA) was performed using DH12S electrocompetent cells following supplier's instructions (Invitrogen). A total of about 8 × 10^5 ^primary recombinant clones were obtained, the average insert size was 1650 bp, and the library was amplified, titred, and stored in 25% of glycerol stocks in a -80°C freezer. When needed, clones were plated on Luria-Bertani (LB) agar medium containing (50 μg/ml chloramphenicol) and grown overnight at 37°C. Then, colonies were randomly picked, inoculated in 384-well microtitre plate (containing LB medium, 50 μg/ml chloramphenicol, and 10 % glycerol), incubated overnight with shaking at 37°C, and stored in a -80°C freezer until further use.

### Plasmid isolation and DNA sequencing

Clones were transferred from 384-well plates to 96-well plates containing LB medium with 50 μg/ml chloramphenicol and grown for about 20 hr prior to plasmid isolation. Plasmid DNA was isolated from 6,528 randomly selected clones using Perfectprep^® ^Plasmid 96 Vac, Direct Bind Kit (Eppendorf). Sequences of cDNAs were sequenced from their 5' end using Big Dye terminator and M13 primer (5'TGTAAAACGACGGCCAGT3') on an ABI 3130*xl *Genetic Analyzer (Applied Biosystems, Foster City, CA) following manufacturer's protocol.

### EST processing, contig assembly and analysis

The chromatogram files were exported to the PHRED program [36] for basecalling and removal of poor quality sequences. Then, vector sequence, adapter region, and poly(A) tails were trimmed from the sequence using Vector *NTI Advance*™ 10 (Invitrogen Corporation, 2005). Trimmed sequences were further screened by including the vector sequence in the contig assembly using the ContigExpress in Vector *NTI Advance*™ 10 (Invitrogen Corporation, 2005) and discarding all ESTs that formed a contig with the vector. High quality ESTs (at least 100 bp after removal of vector sequence, adapter, and poly(A) tail) were then assembled into clusters of contiguous sequences (contigs). Three software programs were used for contig assembly, namely, PHRAP, CAP3 [37], and Vector *NTI Advance*™ 10. After trying the default parameters of the three programs, varying the stringencies of the different parameters, and manually examining the contigs, the results for Vector *NTI *(using more stringent parameters, *i.e*, overlap length cutoff of 50 and overlap percent identity of 90) were retained for further analysis. The ContigExpress module in Vector *NTI Advance*™ 10 also makes use of CAP3 to drive the assembly process (Invitrogen Corporation, 2005). The consensus sequence of each contig and singletons comprised the unique sequences which were compared against the National Center for Biotechnology Information (NCBI) nonredundant protein database using BLASTX. ESTs that did not match sequences in the protein database were further analyzed by searching for similarities at the nucleotide level by using BLASTN. Novel ESTs were also identified by comparison with *C. virginica *EST sequences in dbEST at NCBI using BLASTN. The E-value cutoff was 1e-5.

### Gene ontology annotation

Sequences with BLASTX hits were mapped and annotated according to gene ontology terms (GO) using the program Blast2GO [38]. The distribution of genes in each of the main ontology categories was examined and the percentages of unique sequences in each of the assigned GO terms were computed. In each of the three main categories of GO, namely, biological process, molecular function, and cellular component [39], 100 % was considered as the total number of unique sequences having an assigned GO term. Thus, in each main category, the percentages do not add up to 100% because some deduced proteins have more than one GO category assigned to them [40].

### Bioinformatic mining of microsatellites and SNPs

The set of 4,688 unique sequences was searched for microsatellites using the web-based software, Msatfinder v. 2.0.9 [41]. The thresholds or minimum repeat number used for the search was 8 for dinucleotide microsatellites, 6 for trinucleotide microsatellites, and 5 for tetra- and pentanucloetide microsatellites. Microsatellite-containing ESTs were identified as candidates for marker development if they contained sufficient flanking sequences on either side of the repeats for primer design using Msatfinder. All existing *C. virginica *ESTs in NCBI's dbEST were added to the ESTs generated in this work for detection of putative SNPs using the AutoSNP program with default parameters [42]. Thus, a total of 14,560 *C. virginica *ESTs were searched for putative SNPs.

### Accession numbers

The ESTs generated from this study have been deposited in GenBank and were assigned accession numbers from EH643873 to EH649414.

## Authors' contributions

JQ conducted the major part of this study. SW assisted in data analysis and manuscript preparation. PL and EP constructed the normalized and subtracted cDNA library. JA assisted in EST sequencing. YW, LW, YS and XG prepared the oyster tissue samples and extracted the RNA, and revised the manuscript. RW provided oyster samples and participated in biological studies of the project. ZL supervised the entire study and prepared the manuscript. All authors read and approved the final manuscript.

## Supplementary Material

Additional file 1 *C. virginica *unique sequences with BLASTX hits in the non-redundant protein database. The table includes EST name, accession number, hit ID, BLAST annotation of the top hit, E-value, organism of the top hit, number of GO annotations, and GO IDs Click here for file

Additional file 2 Details of GO annotation. This table provides the detailed information of GO annotation of each contig sequence or EST sequence. Click here for file

Additional file 3 Unigene clusters used for subtraction. This table provides the detailed information of the unigene clusters used for the subtraction during normalized cDNA library construction. Click here for file
